# Supplementary material for: Study of Pressure Distribution in Floor Tiles with Printed P(VDF:TrFE) Sensors for Smart Surface Applications
Source: Sensors (Basel). 2023 Jan 5;23(2):603. doi: 10.3390/s23020603 (PMC9860637; doi:10.3390/s23020603)
Supplement: Supplementary file 1 [file sensors-23-00603-s001.zip › manuscript_v5_SM.pdf]

Supplementary Material

Study of Pressure Distribution in Floor Tiles with Printed P(VDF:TrFE) Sensors for Smart Surface Applications

Asier Alvarez Rueda[1], Philipp Schäffner [1,*], Andreas Petritz [1], Jonas Groten [1], Andreas Tschepp [1], Frank Petersen[2], Martin Zirkl[1], Barbara Stadlober[1]

[1] Joanneum Research Forschungsgesellschaft mbH, Franz-Pichler-Straße 30, 8160 Weiz, Austria
[2] Parador GmbH, Millenkamp 7-8, 48653 Coesfeld, Germany
* Correspondence: philipp.schaeffner@joanneum.at; Tel.: +43-316-876-3107

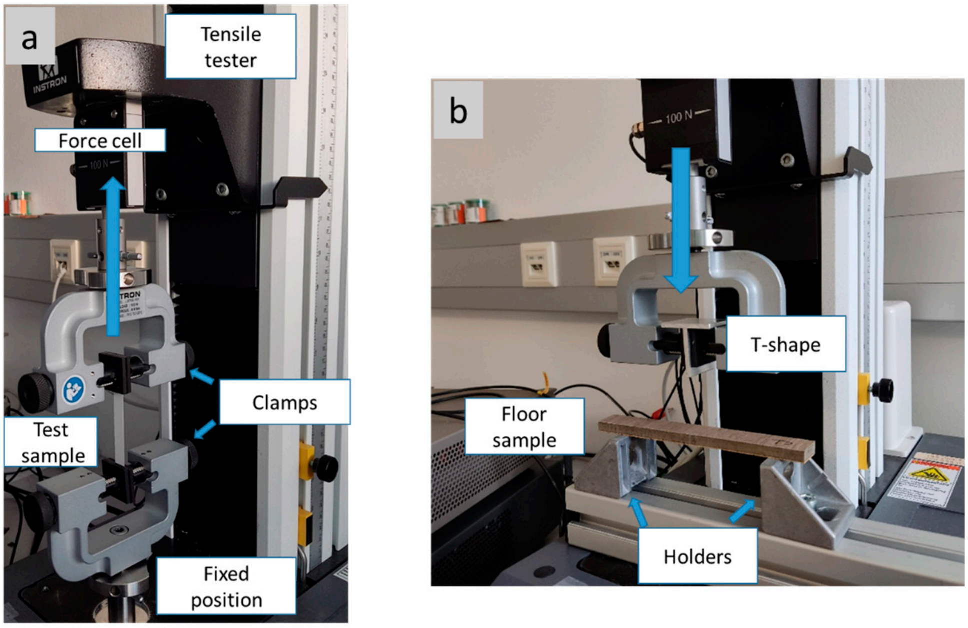

Figure S1. Experimental setups for (**a**) tensile test and (**b**) three point flexural test. In (**a**) the top clamp moves upwards while the force cell registers the required force. In (**b**) the clamp holds a T-shape aluminum profile and moves downwards to apply a force in the middle of the sample.

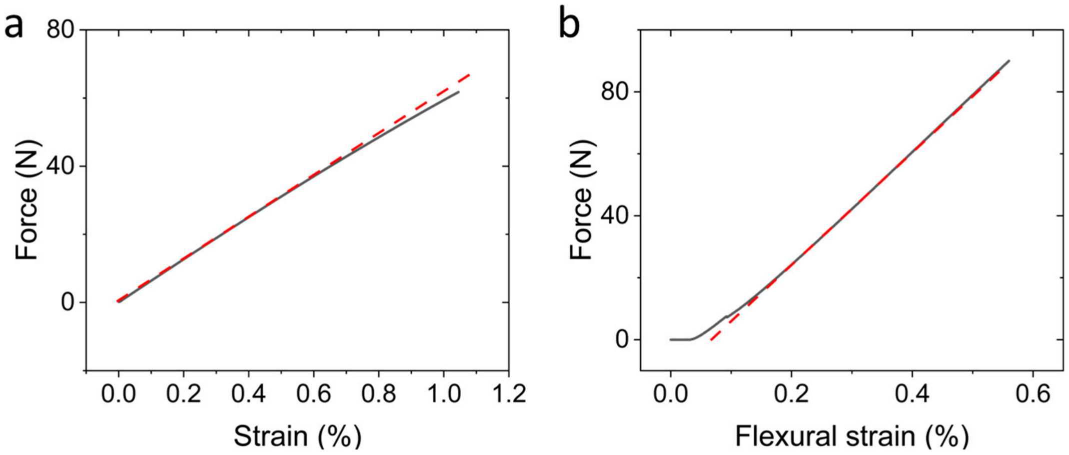

Figure S2. Results of the (**a**) three point flexural test of a parquet sample and (**b**) tensile test of the PET sample. In both cases, a straight line (dashed red) is added as a guide for the eye. We can see a linear behavior in both cases, although for PET the deviation appears starting from a strain of 0.8 %.

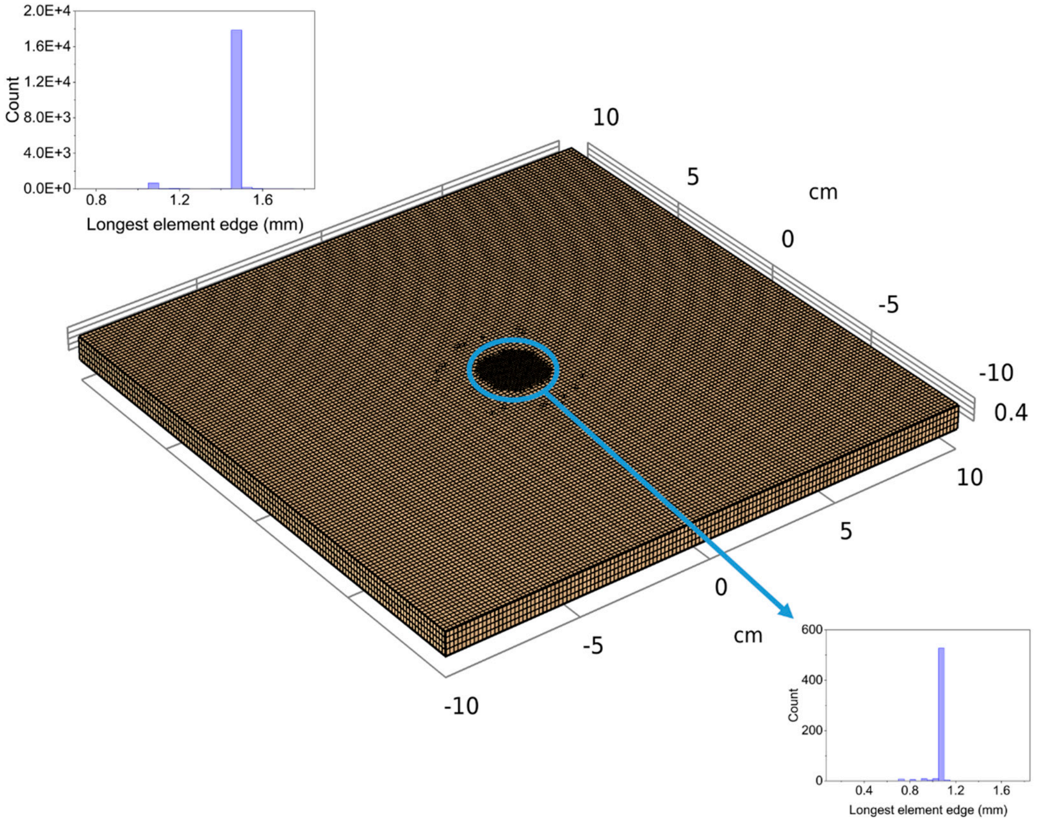

Figure S3. Mesh for the FEM model. We used a higher mesh density in the middle to increase the model's accuracy. This was obtained through an automatic mesh refining step. The histograms show the distribution of elements in the top surface by the length of their longest edge, for the whole mesh (**top left**) and in the refined area (**bottom right**). The mesh is mostly composed by elements with a maximum size of 1.47 mm, and refined elements of 1.07 mm.

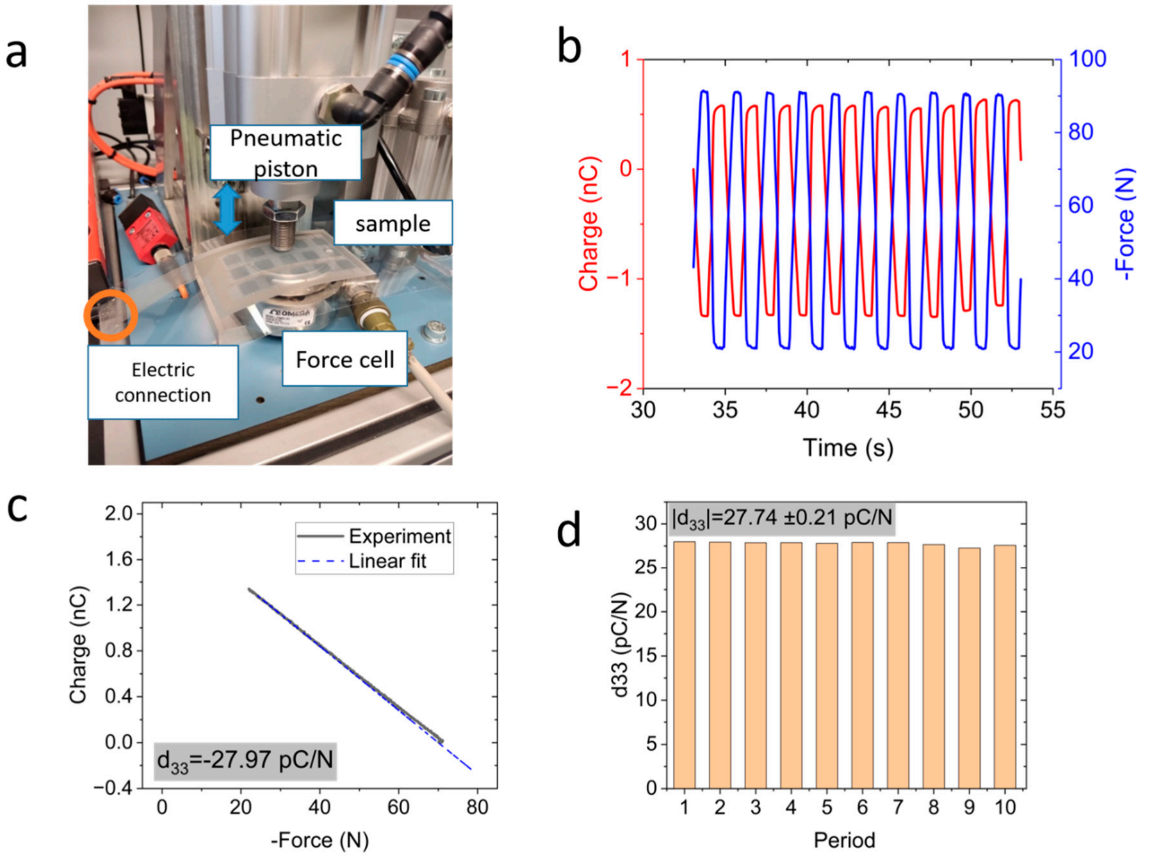

Figure S4. d_{33} measuring experiment for a single pixel: (**a**) Image of experimental setup. As indicated in the text, the piston performs a press-release movement. The sample is then connected to a National Instruments PXI card for current measuring, while the applied force is recorded with a load cell. (**b**) Raw data (short circuit charge and applied force). In order to ensure a stable regime has been reached, press-release periods are performed for 30 s before recording. (**c**) Charge versus force, for a single press movement. A value for d_{33} is obtained through linear regression. (**d**) Fitted d_{33}values for 10 periods. The final result is the average value of said periods, which is indicated above the bars.
